# Supplementary figures and images for: Trichomonas vaginalis: Monolayer and Cluster Formation—Ultrastructural Aspects Using High-Resolution Scanning Electron Microscopy
Source: Pathogens. 2023 Nov 23;12(12):1381. doi: 10.3390/pathogens12121381 (PMC10747464; doi:10.3390/pathogens12121381)

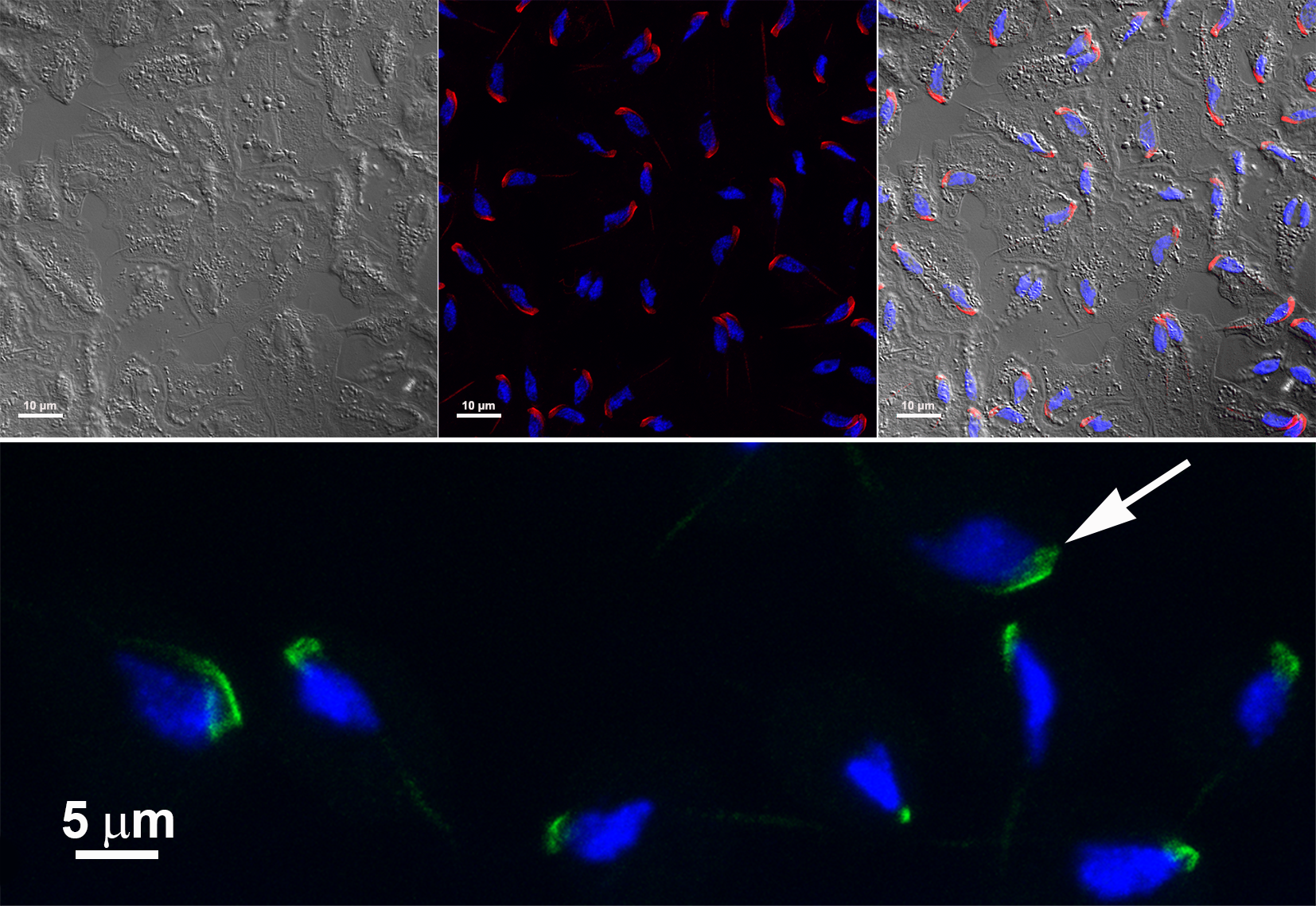

Supplement: Supplementary file 1 [file pathogens-12-01381-s001.zip › Figure S1.tif]
